# Diabetic kidney disease and risk of incident stroke among adults with type 2 diabetes

**DOI:** 10.1186/s12916-022-02317-0

**Published:** 2022-03-29

**Authors:** Arnaud D. Kaze, Bernard G. Jaar, Gregg C. Fonarow, Justin B. Echouffo-Tcheugui

**Affiliations:** 1 Department of Medicine, SOVAH Health, Danville, VA USA; 2 Department of Medicine, Johns Hopkins School of Medicine, Baltimore, MD USA; 3 Welch Welch Center for Prevention, Epidemiology and Clinical Research, Johns Hopkins University, Baltimore, MD USA; 4 Department of Epidemiology, Johns Hopkins Bloomberg School of Public Health, Baltimore, MD USA; 5 Nephrology Center of Maryland, Baltimore, USA; 6 Ahmanson-UCLA Cardiomyopathy Center, Ronald Reagan UCLA Medical Center, Los Angeles, CA USA

**Keywords:** Albuminuria, Diabetic kidney disease, Epidemiology, Glomerular filtration rate, Stroke, Type 2 diabetes

## Abstract

**Background:**

Data on the relations between kidney function abnormalities and stroke in type 2 diabetes are limited. We evaluated the associations of kidney function abnormalities and chronic kidney disease (CKD) stages with incident stroke in a large sample of adults with type 2 diabetes.

**Methods:**

Participants with type 2 diabetes from the Action to Control Cardiovascular Risk in Diabetes (ACCORD) study without history of stroke at baseline were included. Urine albumin-to-creatinine ratio (UACR) and estimated glomerular filtration rate (eGFR) were assessed at baseline. CKD categories were defined according to the KDIGO (Kidney Disease: Improving Global Outcomes) guidelines. Cox proportional hazards regression models were used to compute hazard ratios (HR) and 95% confidence intervals (CI) for stroke in relation to measures of kidney function and CKD categories.

**Results:**

A total of 9170 participants (mean age 62.8 [SD: 6.6] years, 38.2% women, 62.9% white) were included. Over a median follow-up of 4.9 years (interquartile range: 4.0–5.7), 156 participants developed a stroke (incidence rate 3.6/1000 person-years [95% CI 3.0–4.2]). After adjusting for relevant confounders, higher UACR and lower eGFR were each associated with increased risk of stroke. Compared to UACR < 30 mg/g, moderate albuminuria and severe albuminuria were associated with increasing hazards for stroke (HR 1.61 [95% CI 1.12–2.32] and 2.29 [95% CI 1.39–3.80], respectively). Compared to eGFR of ≥ 60 mL/min/1.73 m^2^, decreased eGFR (eGFR < 60 mL/min/1.73 m^2^) was associated with higher risk of stroke (HR 1.50, 95% CI 0.98–2.29). Compared to no CKD, worsening CKD stage was associated with an increasing risk of stroke (HRs of 1.76 [95% CI 1.10–2.83] for CKD G1, 1.77 [95% CI 1.13–2.75] for CKD G2, and 2.03 [95% CI 1.27–3.24] for CKD G3).

**Conclusions:**

In a large sample of adults with type 2 diabetes, increasing albuminuria and worsening stages of early CKD were independently associated with higher risk of incident stroke.

**Trial registration:**

ClinicalTrials.gov. Identifier: NCT00000620.

**Supplementary Information:**

The online version contains supplementary material available at 10.1186/s12916-022-02317-0.

## Background

Atherosclerotic cardiovascular disease (ASCVD) is a significant cause of morbidity and mortality among people with type 2 diabetes [1, 2]. The huge burden of ASCVD in type 2 diabetes, mainly coronary artery disease (CAD) and stroke, constitutes a major public health problem in this population and accounts for significant disability and healthcare costs [3]. Diabetes mellitus is associated with a 3-fold greater age-adjusted risk of stroke and a higher post-stroke mortality [3]. The increased burden of stroke in individuals with type 2 diabetes is driven in large part by the high prevalence of the metabolic syndrome components in this population [3]. However, accruing evidence suggest that other factors such as diabetes-related microvascular complications might play a role [4–7]. Indeed, diabetic retinopathy was found to be associated with higher risk of stroke in people with type 2 diabetes [8]. The impact on stroke of microvascular disease in other vascular territories (such as the kidney) in individuals with type 2 diabetes has received less attention [9–11].

We aimed to assess the relations of abnormalities in urine albumin-to-creatinine ratio (UACR), estimated glomerular filtration rate (eGFR), and earlier stages (G1-G3) of chronic kidney disease (CKD) (KDIGO [Kidney Disease: Improving Global Outcomes] classification) [12] with the risk of stroke in adults with type 2 diabetes, using data from the Action to Control Cardiovascular Risk in Diabetes (ACCORD) study. We hypothesized that kidney function abnormalities and worsening CKD stages would be associated with higher risk of incident stroke.

## Methods

### Study design

The details about the design of the ACCORD study have previously been published [13]. Briefly, ACCORD enrolled 10251 patients with type 2 diabetes with glycated hemoglobin (HbA_1C_) ≥ 7.5% who were either between the ages 40 and 79 years with established cardiovascular disease (CVD) or between the ages of 55 and 79 with evidence of significant atherosclerosis, left ventricular hypertrophy, albuminuria, or ≥ 2 additional CVD risk factors (current smoking, hypertension, dyslipidemia, or obesity). Exclusion criteria included frequent or recent hypoglycemic events, unwillingness to perform home glucose monitoring or self-administer insulin, a serum creatinine > 1.5 mg/dL or other serious illness. Additionally, individuals with CKD stages G4 or G5 were excluded from ACCORD. The 10251 participants were randomly assigned to receive either intensive glucose lowering therapy aiming for HbA_1C_ < 6.0% or standard therapy targeting HbA_1C_ of 7.0 to 7.9%. Participants were further randomized in a double two-by-two factorial design with 4733 individuals randomly assigned to receive intensive blood pressure (BP) control (systolic BP target < 120 mm Hg) or standard treatment (systolic BP < 140 mm Hg); 5518 participants randomly assigned to either fenofibrate or placebo while maintaining adequate low-density lipoprotein (LDL) cholesterol using simvastatin.

For the current analysis, participants with history of prior stroke (*n* = 630) or missing data on UACR/eGFR (*n* = 451) were excluded. After these exclusions, 9170 participants were included in our analyses. The exclusion process is summarized in Additional file 1, Supplementary Fig. S1.

The ACCORD study was conducted in accordance with the principles of the Helsinki declaration. All participants provided written informed consent, and the study protocol was approved by the institutional review board or ethics committee of each participating center.

### Assessment of kidney function abnormalities

Blood and urine samples were collected at baseline from each participant. All urine and serum specimens were analyzed on the day of the sample receipt. Serum and urine creatinine was measured via enzymatic methods on a Roche Double Modular P Analytics automated analyzer. The inter-assay precision coefficients have been reported to be less than 1.4 and less than 2.2% for the % for the high-quality control and low-quality control samples, respectively [14]. eGFR was calculated in mL/min/1.73 m^2^ using the Chronic Kidney Disease Epidemiology Collaboration (CKD-EPI) formula [15]. Urine microalbumin was assayed on spot urine by immunonephelometry on a Siemens BN11 nephelometer. The sensitivity of this assay was 0.16 mg/dL with an inter-assay coefficient of variation of 3.0%, 2.6% and 4.9% for control levels of 0.89 mg/dL, 6.6 mg/dL, and 16.1 mg/dL, respectively. Urinary albumin excretion was estimated as the UACR in mg albumin per g of creatinine.

Kidney function abnormalities were defined as clinically relevant categories. UACR was categorized as normal (UACR< 30 mg/g), moderate albuminuria (UACR 30 to < 300 mg/g), and severe albuminuria (UACR ≥300 mg/g). eGFR was categorized as decreased eGFR (eGFR < 60 mL/min/1.73 m^2^) or normal eGFR (eGFR ≥ 60 mL/min/1.73 m^2^) [12].

CKD stages were defined by categories of eGFR and UACR based on the KDIGO clinical practice guideline as follows: (1) no CKD defined as eGFR ≥ 60 and UACR < 30; (2) CKD G1, as eGFR ≥ 90 and UACR ≥ 30; (3) CKD G2, as eGFR between 60 and 89 and UACR ≥ 30; and (4) CKD G3, as eGFR between 30 and 59 regardless of UACR [12]. Of note, participants with stages G4 and G5 were excluded by design in ACCORD [13].

Additionally, participants were classified according to risk categories defined by the KDIGO guidelines: (1) low risk was defined as eGFR ≥ 60 and UACR < 30; (2) moderate risk, as (45 ≤ eGFR < 60 and UACR < 30) or (eGFR ≥ 60 and 30 ≤ UACR ≤ 300); (3) high risk, as (30 ≤ eGFR < 44 and UACR < 30) or (45 ≤ eGFR < 60 and 30 ≤ UACR ≤ 300) or (eGFR ≥ 60 and UACR > 300); (4) very high risk, as 30 ≤ eGFR < 44 and (30 ≤ UACR ≤ 300) or (30 ≤ eGFR < 60 and UACR> 300) [12].

### Ascertainment of stroke

Participants were followed from enrollment through the onset of stroke, death, or end of study. Stroke cases were ascertained at clinic visits scheduled every 4 months during which participants were asked about emergency room visits and hospitalizations since the previous visit. In case participants did not attend a clinic visit, clinic staff contacted them and conducted events ascertainment via telephone. Stroke was defined as a focal neurologic deficit lasting more than 24 h, with evidence of brain infarction or hemorrhage by computed tomography, magnetic resonance imaging, or autopsy. An expert adjudication committee blinded to the study interventions confirmed the stroke cases’ adjudication [13, 16]. Stroke cases were not further classified into ischemic vs hemorrhagic.

### Covariates

Covariates were selected a priori based on their relationship with kidney function and stroke. They included the following variables collected at baseline: age, sex, race/ethnicity, treatment arm, body mass index (BMI), cigarette smoking status, alcohol intake, BP, use of BP-lowering medications, atrial fibrillation diagnosed on 12-lead electrocardiograms, established ASCVD excluding stroke (history of prior myocardial infarction, coronary revascularization, carotid or peripheral revascularization, or angina), HbA_1C_, duration of diabetes, and lipid variables [13].

### Statistical analyses

The baseline characteristics of study participants were compared by KDIGO CKD categories, as well as UACR and eGFR using the Analysis of Variance or Kruskal-Wallis test for continuous variables depending on their distribution; and the *χ*^2^ test for categorical variables.

Incidence rates (IR) of stroke per 1000 person-years were computed by dividing the number of stroke cases by the person-years estimated from baseline through the earliest of date of stroke’s occurrence, date of death, or study end. The time-to-event distributions for stroke were assessed using the Kaplan-Meier curve and compared by CKD stages using the log-rank test. Cox proportional hazard regression models were used to generate hazard ratios (HRs) and 95% confidence intervals (CIs) for stroke by UACR, eGFR (analyzed as continuous and categorical variables), and CKD stages. For analyses as continuous variables, UACR and eGFR were logarithmically transformed and HRs were reported per standard deviation (SD) increment of the log-transformed value.

We constructed regression models sequentially by first accounting for age, sex, race, and treatment arm (model 1); model 2 accounted for variables in model 1 plus further adjustment for duration of diabetes, hemoglobin A_1C_, cigarette smoking, and alcohol intake; body mass index, total-to-HDL cholesterol, systolic BP, use of BP-lowering medications, and atrial fibrillation; and history of CVD (excluding stroke). Model 3 adjusted for model 2 variables plus use of antiplatelet agents (including aspirin)/anticoagulants, as well as various classes of antihypertensive medications that may affect kidney function such as use of angiotensin-converting enzyme inhibitor (ACEI)/ angiotensin-II receptor blocker (ARB), and diuretics. For analyses of UACR, model 4 further adjusted for baseline eGFR; alternatively, for the assessment of eGFR, model 4 included additional adjustment for baseline UACR.

In sensitivity analyses, we repeated the analyses after excluding participants on fenofibrate as fenofibrate has been shown to increase serum creatinine without causing any alterations in kidney function [17]. Additionally, we tested for statistical interaction of CKD stage by age, sex, race/ethnicity, and treatment arm.

A two-sided *P*-value < 0.05 was considered statistically significant for all analyses. The analyses were performed using STATA 14.2 (Stata, Inc., College Station, TX).

## Results

### Baseline characteristics

A comparison of characteristics of participants excluded (due to prior history of stroke or missing values on kidney function markers, Additional file 1, Supplementary Fig. S1) to those included in the final analytical sample is displayed in Additional file 1, Supplementary Table S1.

A total of 9170 participants were included in the analyses (mean age: 62.8 [SD: 6.6] years, 38.2% women, 62.9% white). Of the entire sample, 62.2% of participants had no CKD; 13.3%, CKD G1; 14.1%, CKD G2; and 10.5%, CKD G3. Compared to those without CKD, participants with higher CKD stages were older, more likely to be women, and had higher systolic BP, total cholesterol, total/HDL cholesterol ratios, and longer durations of diabetes. They were also more likely to have atrial fibrillation, hypertension, and history of CVD at baseline (Table 1). The characteristics of study participants were similar when compared by CKD risk categories (Additional file 1, Supplementary Table S2) as well as UACR and eGFR categories (Additional file 1, Supplementary Table S3).

**Table 1:** Baseline characteristics of participants by CKD stage

Characteristics	Whole sample	KDIGO CKD stages				***P*** value*
		No CKD	CKD G1	CKD G2	CKD G3	
*N*	9170	5703	1218	1290	959	…
Age, years	62.8 (6.6)	62.1 (6.2)	60.0 (5.3)	65.2 (7.0)	67.4 (6.6)	< 0.001
Women, %	38.2	39.8	36.4	27.4	45.4	< 0.001
Race/ethnicity, %						< 0.001
*White*	62.9	63.6	57.0	61.7	67.9	
*Black*	18.6	18.3	22.3	20.8	14.4	
*Hispanic*	7.0	6.8	7.9	7.1	6.9	
*Other*	11.3	11.3	12.9	10.4	10.8	
Intensive glycemic management arm, %	50.3	50.2	49.8	50.1	51.7	0.817
Body mass index, kg/m^2^	32.3 (5.4)	32.2 (5.3)	32.9 (5.6)	32.2 (5.5)	32.3 (5.5)	< 0.001
Current smoking, %	13.8	13.1	20.9	14.2	8.2	< 0.001
Alcohol drinking, %	24.4	25.2	25.6	24.9	17.2	< 0.001
Systolic BP, mm Hg	136.3 (17.0)	133.6 (15.8)	130.9 (17.1)	142.0 (18.4)	138.6 (18.0)	< 0.001
Diastolic BP, mm Hg	74.9 (10.6)	74.9 (10.3)	77.4 (10.5)	74.9 (11.2)	71.9 (11.2)	< 0.001
Use of BP-lowering drug, %	83.4	80.3	84.2	90.1	92.0	< 0.001
Use of ACEI/ARB, %	69.1	65.9	71.5	75.4	76.0	< 0.001
Use of diuretics, %	36.1	32.3	31.0	43.4	55.5	< 0.001
Use of antiplatelets/ anticoagulants, %	57.5	56.4	54.8	60.7	62.5	< 0.001
Atrial fibrillation, %	1.2	0.7	1.1	2.6	2.7	< 0.001
History of CVD	31.3	27.9	33.7	37.1	40.2	< 0.001
Hemoglobin A_1C_, %	8.3 (1.1)	8.2 (1.0)	8.6 (1.1)	8.4 (1.1)	8.3 (1.2)	< 0.001
Duration of diabetes, years	9.0 (5.0–15.0)	8.0 (4.5–14.0)	10.0 (5.0–15.0)	11.0 (7.0–18.0)	11.0 (6.0–19.0)	< 0.001
Total cholesterol, mg/dL	183.5 (41.7)	182.5 (40.2)	188.5 (47.3)	181.9 (41.5)	185.6 (42.8)	< 0.001
HDL-cholesterol, mg/dL	41.8 (11.6)	42.3 (11.5)	40.8 (11.5)	41.3 (11.9)	41.0 (11.4)	< 0.001
LDL-cholesterol, mg/dL	105.0 (34.0)	105.1 (33.2)	105.0 (35.7)	104.2 (34.9)	105.8 (34.7)	0.721
Total/HDL-cholesterol ratio	4.7 (1.7)	4.6 (1.6)	4.9 (2.0)	4.7 (1.6)	4.8 (1.7)	< 0.001
UACR, mg/g	14.0 (7.0–45.0)	9.0 (6.0–14.0)	78.0 (45.0–192.0)	97.0 (51.0–239.0)	23.0 (9.0–106.0)	< 0.001
eGFR, mL/min/1.73 m^2^	86.7 (71.8–96.7)	90.4 (77.9–97.6)	97.7 (93.9–104.2)	75.4 (67.8–82.5)	52.7 (47.3–56.7)	< 0.001

*P values are for comparisons between CKD stages using the analysis of variance (ANOVA) for continuous variables with a normal distribution, the Kruskal-Wallis test for continuous variables with a skewed distribution, and the *χ*^2^ test for categorical variables. Data are mean (standard deviation), median (interquartile range), or proportion (%) unless otherwise indicated. CKD was classified according to the KDIGO clinical practice guidelines as follows: 1) No CKD defined as eGFR ≥ 60 and UACR < 30; CKD G1, as eGFR ≥ 90 and UACR ≥ 30; CKD G2, as eGFR between 60 and 89 and UACR ≥ 30; CKD G3, as eGFR between 30 and 59 regardless of UACR. *ACEI* indicates angiotensin-converting enzyme inhibitors, *ARB* angiotensin-II receptor blockers, *BP* blood pressure, *CKD* chronic kidney disease, *CVD* cardiovascular disease, *eGFR* estimated glomerular filtration rate in mL/min/1.73 m^2^, *HDL* high-density lipoprotein, *KDIGO* Kidney Disease: Improving Global Outcomes, *LDL* low-density lipoprotein, *UACR* urine albumin-to-creatinine ratio in mg/g

### Incident stroke by UACR and eGFR levels

Over a median follow-up of 4.9 years (interquartile range: 4.0–5.7), a total of 156 participants experienced a stroke event (incidence rate 3.6/1000 person-years [95% CI 3.0–4.2]).

After multivariable adjustment, UACR was associated with elevated risks of incident stroke. Compared to normal UACR (Table 2), moderate albuminuria and severe albuminuria were each associated with greater hazards of stroke (HRs 1.61 [95% CI 1.12–2.32] and 2.29 [95% CI 1.39–3.80), respectively). Each 1-SD increase in log-transformed UACR was associated with a 44% higher risk of stroke (HR 1.44, 95% CI 1.24–1.68).

**Table 2:** Rates and hazard ratios of incident stroke by measures of kidney function

Kidney function measures	No events/no at risk	P-years	Rate/1000 p-years	Model 1		Model 2		Model 3		Model 4	
				HR (95% CI)	***P***	HR (95% CI)	***P***	HR (95% CI)	***P***	HR (95% CI)	***P***
**Albuminuria**											
***Normal***	74/6240	30112.4	2.5 (2.0–3.1)	1 (reference)	…	1 (reference)		1 (reference)		1 (reference)	
***Moderate***	57/2337	11077.5	5.1 (4.0–6.7)	1.99 (1.41–2.83)	< 0.001	1.63 (1.13–2.33)	0.009	1.62 (1.13–2.33)	0.009	1.61 (1.12–2.32)	0.010
***Severe***	25/593	2735.2	9.1 (6.2–13.5)	3.69 (2.34–5.81)	< 0.001	2.47 (1.50–4.06)	< 0.001	2.43 (1.47–3.99)	0.001	2.29 (1.39–3.80)	0.001
***Ln (UACR)***	156/9170	43925.0	3.6 (3.0–4.2)	1.64 (1.44–1.87)	< 0.001	1.47 (1.27–1.70)	< 0.001	1.47 (1.26–1.70)	< 0.001	1.44 (1.24–1.68)	< 0.001
**eGFR, mL/min/1.73 m**^**2**^											
***Normal***	123/8211	39205.5	3.1 (2.6–3.7)	1 (reference)	…	1 (reference)	…	1 (reference)	…	1 (reference)	
***Decreased***	33/959	4719.5	7.0 (5.0–9.8)	1.83 (1.22–2.74)	0.004	1.60 (1.05–2.43)	0.030	1.55 (1.02–2.37)	0.042	1.50 (0.98–2.29)	0.060
***Ln (eGFR)***	156/9170	43925.0	3.6 (3.0–4.2)	1.26 (1.09–1.47)	0.003	1.22 (1.04–1.42)	0.014	1.21 (1.03–1.41)	0.020	1.18 (1.01–1.38)	0.034

Albuminuria was classified based on UACR as normal (UACR< 30 mg/g), moderate (UACR 30 to < 300 mg/g), and severe (UACR ≥ 300 mg/g). eGFR was categorized as decreased (eGFR < 60 mL/min/1.73 m^2^) or normal (eGFR ≥ 60 mL/min/1.73 m^2^)

Model 1 adjusted for age, sex, race, and treatment arm; model 2, model 1 variables plus duration of diabetes, hemoglobin A_1C_, cigarette smoking, alcohol intake; body mass index, total-to-HDL cholesterol, systolic BP, use of BP-lowering medications, atrial fibrillation; history of CVD (excluding stroke) at baseline; model 3, model 2 variables plus use of antiplatelet agents (including aspirin)/ anticoagulants, diuretics, ACEI/ARB; model 4, model 3 variables plus eGFR (when assessing UACR) or UACR (when assessing eGFR). For continuous measures, hazard ratios are reported per 1-SD increment of the natural logarithm (ln) of UACR (SD = 1.46) or 1-SD decrease of the natural logarithm (ln) of eGFR (SD = 0.23).*ACEI* indicates angiotensin-converting enzyme inhibitors, *ARB* angiotensin-II receptor blockers, *BP* blood pressure, *CI* confidence interval, *CVD* cardiovascular disease, *eGFR* estimated glomerular filtration rate, *HDL* high-density lipoprotein, *HR* hazard ratio, *p-years* person-years, *SD* standard deviation, *UACR* urine albumin-creatinine ratio

Participants with decreased eGFR (eGFR < 60 mL/min/1.73 m^2^) had a 50% greater risk of stroke (HR 1.50, 95% CI 0.98–2.29) compared to those with normal eGFR (eGFR ≥ 60 mL/min/1.73 m^2^). Each 1-SD decrease in log-transformed eGFR was associated with an 18% increased risk of stroke (HR 1.18, 95% CI 1.01–1.38).

Compared with participants with both normal UACR and eGFR (Table 3), those with UACR ≥ 30 mg/g and eGFR < 60 mL/min/1.73 m^2^ had increased hazards of stroke (HR 2.54, 95% CI 1.45–4.43).

**Table 3:** Rates and hazard ratios of incident stroke by combined measures of kidney function

Measures of kidney function		No events/no at risk	P-years	Rate/1000 p-years	Model 1		Model 2		Model 3	
					HR (95% CI)	***P***	HR (95% CI)	***P***	HR (95% CI)	***P***
**UACR**	**eGFR**									
**< 30**	**≥ 60**	61/5703	27414.3	2.2 (1.7–2.9)	1 (reference)		1 (reference)		1 (reference)	
**< 30**	**< 60**	13/537	2698.0	4.8 (2.8–8.3)	1.76 (0.95–3.26)	0.070	1.64 (0.88–3.06)	0.117	1.59 (0.86–2.97)	0.142
**≥ 30**	**≥ 60**	62/2508	11791.2	5.3 (4.1–6.7)	2.30 (1.61–3.28)	< 0.001	1.80 (1.24–2.61)	0.002	1.79 (1.23–2.60)	0.002
**≥ 30**	**< 60**	20/422	2021.5	9.9 (6.4–15.3)	3.66 (2.17–6.17)	< 0.001	2.61 (1.50–4.55)	0.001	2.54 (1.45–4.43)	0.001

Model 1 adjusted for age, sex, race, and treatment arm; model 2, model 1 variables plus duration of diabetes, hemoglobin A_1C_, cigarette smoking, alcohol intake; body mass index, total-to-HDL cholesterol, systolic BP, use of BP-lowering medications, atrial fibrillation; history of CVD (excluding stroke) at baseline; model 3, model 2 variables plus use of antiplatelet agents (including aspirin)/ anticoagulants, diuretics, ACEI/ARB

*ACEI* indicates angiotensin-converting enzyme inhibitors, *ARB* angiotensin-II receptor blockers, *BP* blood pressure, *CI* confidence interval, *CVD* cardiovascular disease, *eGFR* estimated glomerular filtration rate, *HDL* high-density lipoprotein, *HR* hazard ratio, *p-years* person-years, *UACR* urine albumin-creatinine ratio

### Incident stroke by KDIGO CKD categories

**Fig. 1:**
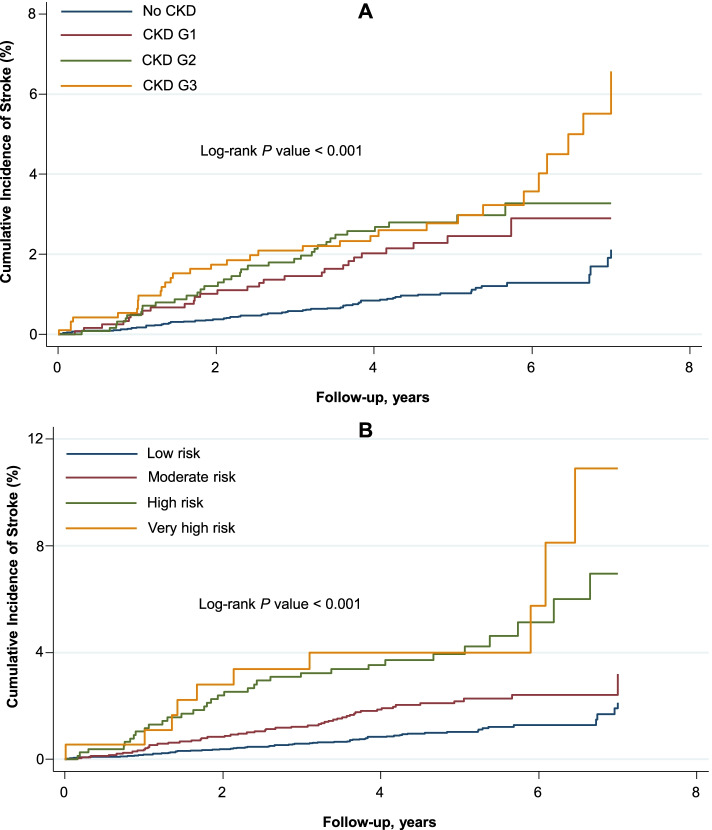
Cumulative Incidence of Stroke by CKD stage (**A**) and CKD risk category (**B**). CKD was classified according to the KDIGO clinical practice guidelines: (1) no CKD defined as eGFR ≥ 60 and UACR < 30; (2) CKD G1, as eGFR ≥ 90 and UACR ≥ 30; (3) CKD G2, as eGFR between 60 and 89 and UACR ≥ 30; (4) CKD G3, as eGFR between 30 and 59 regardless of UACR. CKD risk categories were defined by eGFR and UACR as follows: (1) low risk, as eGFR ≥ 60 and UACR < 30; (2) moderate risk, as (45 ≤ eGFR < 60 and UACR < 30) or (eGFR ≥ 60 and 30 ≤ UACR ≤ 300); (3) high risk, as (30 ≤ eGFR < 44 and UACR< 30) or (45 ≤ eGFR < 60 and 30 ≤ UACR ≤ 300) or (eGFR ≥ 60 and UACR > 300); (4) very high risk, as (30 ≤ eGFR < 44 and 30 ≤ UACR ≤ 300) or (30 ≤ eGFR < 60 and UACR > 300). CKD indicates chronic kidney disease; eGFR, estimated glomerular filtration rate; KDIGO, Kidney Disease: Improving Global Outcomes; UACR, urine albumin-creatinine ratio

Participants with higher CKD stages had increased cumulative incidence of stroke as compared to those with lower stages (Fig. 1, *P-*value-log rank < 0.001).

Compared to the absence of CKD, increasing CKD stages were associated with greater risk of stroke; the adjusted HRs were 1.76 (95% CI 1.10–2.83) for CKD G1, 1.77 (95% CI 1.13–2.75) for CKD G2, and 2.03 (95% CI 1.27–3.24) for CKD G3. Additionally, compared to the low-risk stage, the HRs for incident stroke were 1.55 (95% CI 1.05–2.27) for the moderate risk stage, 2.56 (95% CI 1.61–4.07) for the high-risk stage, and 2.86 (95% CI 1.41–5.80) for the very high-risk stage (Table 4).

**Table 4:** Rates and hazard ratios of incident stroke by KDIGO CKD categories

CKD classification	No events/no at risk	P-years	Rate/1000 p-years	Model 1		Model 2		Model 3	
				HR (95% CI)	***P***	HR (95% CI)	***P***	HR (95% CI)	***P***
**CKD stage**									
***No CKD***	61/5703	27414.3	2.2 (1.7–2.9)	1 (reference)	…	1 (reference)	**…**	1 (reference)	
***CKD G1***	27/1218	5701.1	4.7 (3.2–6.9)	2.27 (1.44–3.59)	< 0.001	1.76 (1.10–2.83)	0.019	1.76 (1.10–2.83)	0.020
***CKD G2***	35/1290	6090.1	5.7 (4.1–8.0)	2.31 (1.51–3.53)	< 0.001	1.79 (1.15–2.79)	0.010	1.77 (1.13–2.75)	0.012
***CKD G3***	33/959	4719.5	7.0 (5.0–9.8)	2.57 (1.65–4.02)	< 0.001	2.09 (1.31–3.33)	0.002	2.03 (1.27–3.24)	0.003
**CKD risk categories**									
***Low risk***	61/5703	27414.3	2.2 (1.7–2.9)	1 (reference)	…	1 (reference)	**…**	1 (reference)	
***Moderate risk***	52/2484	11876.6	4.4 (3.3–5.7)	1.84 (1.27–2.68)	0.001	1.56 (1.06–2.29)	0.023	1.55 (1.05–2.27)	0.026
***High risk***	33/797	3719.8	8.9 (6.3–12.5)	3.62 (2.35–5.57)	< 0.001	2.62 (1.65–4.16)	< 0.001	2.56 (1.61–4.07)	< 0.001
***Very high risk***	10/186	914.4	10.9 (5.9–20.3)	4.22 (2.14–8.32)	< 0.001	2.95 (1.46–5.96)	0.003	2.86 (1.41–5.80)	0.004

CKD was classified according to the KDIGO clinical practice guidelines as follows: (1) no CKD defined as eGFR ≥ 60 and UACR < 30; (2) CKD G1, as eGFR ≥ 90 and UACR ≥ 30; (3) CKD G2, as eGFR between 60 and 89 and UACR ≥ 30; (4) CKD G3, as eGFR between 30 and 59 regardless of UACR

CKD risk categories were defined by eGFR and UACR as follows: (1) low risk, as eGFR ≥ 60 and UACR < 30; (2) moderate risk, as (45 ≤ eGFR < 60 and UACR < 30) or (eGFR ≥ 60 and 30 ≤ UACR ≤ 300); (3) high risk, as (30 ≤ eGFR < 44 and UACR < 30) or (45 ≤ eGFR < 60 and 30 ≤ UACR ≤ 300) or (eGFR ≥ 60 and UACR > 300); (4) very high risk, as (30 ≤ eGFR < 44 and 30 ≤ UACR ≤ 300) or (30 ≤ eGFR < 60 and UACR> 300)

Model 1 adjusted for age, sex, race, and treatment arm; model 2, model 1 variables plus duration of diabetes, hemoglobin A_1C_, cigarette smoking, alcohol intake; body mass index, total-to-HDL cholesterol, systolic BP, use of BP-lowering medications, atrial fibrillation; history of CVD (excluding stroke) at baseline; model 3, model 2 variables plus use of antiplatelet agents (including aspirin)/ anticoagulants, diuretics, ACEI/ARB.

*ACEI* indicates angiotensin-converting enzyme inhibitors, *ARB* angiotensin-II receptor blockers, *BP* blood pressure, *CKD* chronic kidney disease, *CI* confidence interval, *CVD* cardiovascular disease, *eGFR* estimated glomerular filtration rate, *HDL* high-density lipoprotein, *HR* hazard ratio, *KDIGO* Kidney Disease: Improving Global Outcomes, *p-years* person-years, *UACR* urine albumin-creatinine ratio

We conducted sensitivity analyses by restricting the analytical sample to participants not on fenofibrate as it has been shown to elevate serum creatinine without altering kidney function. These did not materially affect the magnitude or significance of the results (Additional file 1, Supplementary Tables S4 & S5).

**Fig. 2:**
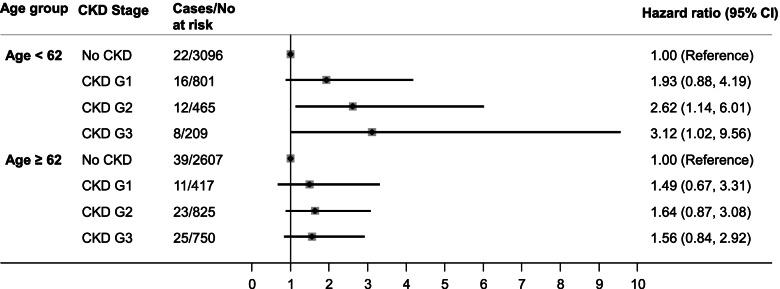
Hazard ratios for incident stroke by age group and CKD stage. Hazard ratios are adjusted for sex, race, treatment arm, duration of diabetes, hemoglobin A_1C_, cigarette smoking, alcohol intake; body mass index, total-to-HDL cholesterol, systolic BP, use of BP-lowering medications, atrial fibrillation and history of cardiovascular disease, use of antiplatelet agents (including aspirin)/ anticoagulants, diuretics, ACEI/ARB. CKD was classified according to the KDIGO clinical practice guidelines: (1) no CKD defined as eGFR ≥ 60 and UACR < 30; (2) CKD G1, as eGFR ≥ 90 and UACR ≥ 30; (3) CKD G2, as eGFR between 60 and 89 and UACR ≥ 30; (4) CKD G3, as eGFR between 30 and 59 regardless of UACR. ACEI indicates angiotensin-converting enzyme inhibitors; ARB, angiotensin-II receptor blockers; BP, blood pressure; CKD, chronic kidney disease; eGFR, estimated glomerular filtration rate; HDL, high-density lipoprotein; KDIGO, Kidney Disease: Improving Global Outcomes; UACR, urine albumin-creatinine ratio

Additionally, we tested for statistical interaction of CKD stage by age, sex, race/ethnicity, and treatment arm. We found statistically significant evidence of interaction by age (*P* = 0.035), but not sex (*P* = 0.400), race/ethnicity (*P* = 0.289), or treatment arm (*P* = 0.342). Since we observed statistical interaction by age, we performed additional analyses stratified into two age groups using the median age (62 years) as the cutoff. The association between CKD stage and stroke was stronger among younger participants (age < 62) whereas no significant association was observed among those 62 or older (Fig. 2).

## Discussion

We evaluated the associations of kidney function abnormalities and CKD stages with incident stroke in adults with type 2 diabetes enrolled in the ACCORD study and made several observations. First, the higher the albuminuria level, the greater was the risk of stroke. Second, decreased eGFR was related to a greater risk of stroke. Third, worsening CKD stages defined by the KDIGO criteria were associated with greater risk of stroke.

Our study has unique features, in that it assesses the impact of several CKD classification schemes on the risks of incident stroke using a prospective study design of adults with type 2 diabetes. A recent systematic review and meta-analysis identified a gap in this area by showing that most studies of the association between CKD and incident stroke did not focus on people with type 2 diabetes [9], and the few studies with a focus on diabetes were limited by a retrospective design [18, 19], a short duration of follow-up [20], a hospital-based setting [20], or the lack of diversity in their study samples [18–20]. Our results are however consistent with prior reports of a positive relation between markers of kidney disease and adverse cardiovascular events both from the general population, as well as people with diabetes mellitus [9, 18–26]. Indeed, in a retrospective analysis of 653 Japanese individuals with type 2 diabetes, participants with CKD stages 2 and 3 had a higher risk of stroke compared to those without CKD. In the same cohort, the incidence of stroke was elevated among participants with kidney function abnormalities assessed in isolation (reduced eGFR as well as increased urinary excretion) [18]. In another cohort of Chinese patients with type 2 diabetes, lower eGFR was independently associated with higher risks of recurrent stroke or transient ischemic attacks [20]. Likewise, decreasing eGFR was also found to be related to increasing hazards of cerebrovascular events in a large primary care database of patients with type 2 diabetes in the UK [19].

While the exact mechanisms linking kidney function abnormalities and incident stroke are incompletely understood, a few hypotheses can be formulated. First, increased urinary albumin excretion has been suggested to be marker of vascular dysfunction and may in fact reflect a widespread vascular process involving the cerebral vasculature, independently of diabetes or hypertension, as shown in studies of people without hypertension or diabetes [27–29]. Additionally, albuminuria may be a reflection of other widespread derangements such as microinflammation, endothelial dysfunction, oxidative stress, and elevated aortic pressures, which are known to increase the risk of atherosclerotic CVD [30]. Moreover, unifying pathways linking microvascular and macrovascular events in diabetes mellitus have been shown [31, 32]. These include the formation of advanced glycation end products from hyperglycemia induced nonenzymatic glycation of proteins. These products may deposit in blood vessel walls, forming cross-links in the extracellular matrix and resulting in several pathological changes including glomerulosclerosis, accelerated atherosclerosis, and thrombosis [31–33].

Our findings should be interpreted in the context of a few limitations. First, patients with advanced CKD (stages G4 and G5) were excluded from ACCORD; hence, the results do not capture the entire spectrum of CKD and are not generalizable to people with advanced CKD. Second, kidney function abnormalities were diagnosed using only one timepoint; while this is commonly performed in large epidemiological studies of CKD [34], this may have led to an overestimation of the true prevalence of CKD in our sample. Third, the ACCORD study did not record data on stroke type (ischemic vs hemorrhagic); hence, we could not evaluate the effect of kidney measures’ abnormalities on stroke type. Finally, our analysis was observational; hence, there remains a possibility of residual confounding. These limitations notwithstanding, this study has several strengths. These include the use of a large and diverse sample of adults with type 2 diabetes, the standardized adjudication of stroke events, and the rigorous evaluation of the various aspects of CKD and its severity using multiple classifications, as well as the rigorous adjustment of potential confounders including diabetic duration and atrial fibrillation.

## Conclusions

In conclusion, in a large and diverse cohort of adults with type 2 diabetes, higher UACR, decreased eGFR, and worsening CKD stages were associated with increased risk of stroke, independently of other stroke risk factors. These data support the notion that the prevention of CKD and its progression may help mitigate the risk of stroke in people with type 2 diabetes. Our findings have several research and public health implications for people with type 2 diabetes. Diabetes mellitus represents the primary cause of CKD in the US [35]. Our findings underscore the notion that strategies designed to prevent the onset and progression of CKD in type 2 diabetes may help mitigate the burden of stroke and its associated disability in this population. Further research is needed to clarify the mechanisms linking measures of kidney disease to stroke and develop strategies targeting both CKD and stroke prevention.

## Supplementary Information

**Additional file 1: Supplementary Fig. S1.** Exclusion criteria for examining the association of diabetic nephropathy and incident stroke among participants enrolled in ACCORD. **Supplementary Table S1.** Comparison of baseline characteristics of Included vs excluded participants. **Supplementary Table S2.** Baseline Characteristics of Participants by CKD risk categories. **Supplementary Table S3.** Baseline Characteristics of Participants by UACR and eGFR. **Supplementary Table S4.** Hazard ratios of incident stroke by combined measures of kidney function after Exclusion of Participants on Fenofibrate. **Supplementary Table S5.** Hazard ratios of incident stroke by KDIGO CKD Categories after Exclusion of Participants on Fenofibrate.

## Data Availability

The data that support the findings of this study are available from the US National Heart, Lung, and Blood Institute (NHLBI) repository (BioLINCC) but restrictions apply to the availability of these data. Data are however available from the corresponding author upon reasonable request and with permission of the National Heart, Lung, and Blood (NHLBI).

## Abbreviations

ACCORD: Action to Control Cardiovascular Risk in Diabetes
ASCVD: Atherosclerotic cardiovascular disease
BP: Blood pressure
CAD: Coronary artery disease
CI: Confidence interval
CKD: Chronic kidney disease
CVD: Cardiovascular disease
eGFR: Estimated glomerular filtration rate
HDL: High-density lipoprotein
HR: Hazard ratio
IR: Incidence rate
KDIGO: Kidney Disease: Improving Global Outcomes
UACR: Urine albumin-creatinine ratio
