# Supplementary material for: Diabetic kidney disease and risk of incident stroke among adults with type 2 diabetes
Source: BMC Med. 2022 Mar 29;20:127. doi: 10.1186/s12916-022-02317-0 (PMC8962078; doi:10.1186/s12916-022-02317-0)
Supplement: Supplementary file 1 — Additional file 1: Supplementary Fig. S1. Exclusion criteria for examining the association of diabetic nephropathy and incident stroke among participants enrolled in ACCORD. Supplementary Table S1. Comparison of baseline characteristics of Included vs excluded participants. Supplementary Table S2. Baseline Characteristics of Participants by CKD risk categories. Supplementary Table S3. Baseline Characteristics of Participants by UACR and eGFR. Supplementary Table S4. Hazard ratios of incident stroke by combined measures of kidney function after Exclusion of Participants on Fenofibrate. Supplementary Table S5. Hazard ratios of incident stroke by KDIGO CKD Categories after Exclusion of Participants on Fenofibrate. [file 12916_2022_2317_MOESM1_ESM.docx]

**DATA SUPPLEMENT**

Participants enrolled in ACCORD (N=10251)

**Exclusions**

- History of stroke at baseline (N=630)
- Missing data on UACR or eGFR (N=451)

Final sample (N=9170)

**Supplementary Figure S1. Exclusion criteria for examining the association of diabetic nephropathy and incident stroke among participants enrolled in ACCORD**

ACCORD indicates Action to Control Cardiovascular Risk in Diabetes; eGFR, estimated glomerular filtration rate; UACR, urine albumin-to-creatinine ratio.

**Supplementary Table S1. Comparison of baseline characteristics of Included vs excluded participants**

|  | **Included** | **Excluded** | ***P*-value** |
| --- | --- | --- | --- |
| N | 9170 | 1081 | … |
| Age, years | 62.8 (6.6) | 62.7 (7.0) | 0.602 |
| Women, % | 38.2 | 41.6 | 0.028 |
| Race/ethnicity, % |  |  | 0.006 |
| White | 62.9 | 57.8 |  |
| Black | 18.6 | 21.6 |  |
| Hispanic | 7.0 | 8.7 |  |
| Other | 11.3 | 11.8 |  |
| Intensive glycemia arm, % | 50.3 | 47.8 | 0.126 |
| Body mass index, kg/m^2^ | 32.3 (5.4) | 31.6 (5.4) | <0.001 |
| Current smoking, % | 13.8 | 15.4 | 0.155 |
| Alcohol drinking, % | 24.4 | 19.9 | 0.001 |
| Systolic BP, mm Hg | 136.3 (17.0) | 137.0 (18.0) | 0.212 |
| Diastolic BP, mm Hg | 74.9 (10.6) | 74.4 (10.8) | 0.096 |
| Use of BP-lowering drug, % | 83.4 | 85.1 | 0.155 |
| Atrial fibrillation, % | 1.2 | 1.3 | 0.835 |
| History of CVD | 31.3 | 68.7 | <0.001 |
| Hemoglobin A_1C_, % | 8.3 (1.1) | 8.3 (1.0) | 0.176 |
| Duration of diabetes, years | 9.0 (5.0-15.0) | 10.0 (5.0-17.0) | <0.001 |
| Total cholesterol, mg/dL | 183.5 (41.7) | 181.3 (43.3) | 0.107 |
| HDL-cholesterol, mg/dL | 41.8 (11.6) | 42.5 (12.2) | 0.066 |
| LDL-cholesterol, mg/dL | 105.0 (34.0) | 103.7 (33.5) | 0.219 |
| Total/HDL-cholesterol ratio | 4.7 (1.7) | 4.6 (1.7) | 0.040 |

Data are mean (standard deviation), median (interquartile range), or proportion (%) unless otherwise indicated.

BP indicates blood pressure; CVD, cardiovascular disease; eGFR, estimated glomerular filtration rate; HDL, high-density lipoprotein; LDL, low-density lipoprotein; UACR, urine albumin-creatinine ratio.

**Supplementary Table S2. Baseline Characteristics of Participants by CKD risk categories**

| **Characteristics** | **Whole sample** |  | **KDIGO CKD risk categories** | | | | ***P* value^*^** |
| --- | --- | --- | --- | --- | --- | --- | --- |
|  |  |  | **Low risk** | **Moderate risk** | **High risk** | **Very high risk** |  |
| N | 9170 |  | 5703 | 2484 | 797 | 186 |  |
| Age, years | 62.8 (6.6) |  | 62.1 (6.2) | 63.6 (6.9) | 64.7 (7.1) | 66.5 (7.4) | <0.001 |
| Women, % | 38.2 |  | 39.8 | 33.4 | 39.7 | 46.2 | <0.001 |
| Race/ethnicity, % |  |  |  |  |  |  | 0.138 |
| *White* | 62.9 |  | 63.6 | 62.4 | 59.4 | 64.0 |  |
| *Black* | 18.6 |  | 18.3 | 19.7 | 20.6 | 13.4 |  |
| *Hispanic* | 7.0 |  | 6.8 | 7.3 | 7.0 | 9.1 |  |
| *Other* | 11.3 |  | 11.3 | 10.7 | 13.1 | 13.4 |  |
| Intensive glycemic management arm, % | 50.3 |  | 50.2 | 50.4 | 50.1 | 52.7 | 0.923 |
| Body mass index, kg/m^2^ | 32.3 (5.4) |  | 32.2 (5.3) | 32.5 (5.5) | 32.3 (5.5) | 32.4 (5.9) | 0.103 |
| Current smoking, % | 13.8 |  | 13.1 | 15.3 | 14.3 | 11.8 | 0.042 |
| Alcohol drinking, % | 24.4 |  | 25.2 | 24.7 | 19.8 | 14.5 | <0.001 |
| Systolic BP, mm Hg | 136.3 (17.0) |  | 133.6 (15.8) | 139.0 (17.2) | 144.8 (19.0) | 146.4 (18.3) | <0.001 |
| Diastolic BP, mm Hg | 74.9 (10.6) |  | 74.9 (10.3) | 75.1 (10.9) | 74.8 (11.8) | 73.2 (11.9) | 0.119 |
| Use of BP-lowering drug, % | 83.4 |  | 80.3 | 87.6 | 90.0 | 94.6 | <0.001 |
| Use of ACEI/ARB, % | 69.1 |  | 65.9 | 73.2 | 76.4 | 77.9 | <0.001 |
| Use of diuretics, % | 36.1 |  | 32.3 | 39.4 | 48.2 | 58.1 | <0.001 |
| Use of Antiplatelets/ anticoagulants, % | 57.5 |  | 56.4 | 59.4 | 59.1 | 55.4 | 0.055 |
| Atrial fibrillation, % | 1.2 |  | 0.7 | 1.9 | 2.4 | 3.2 | <0.001 |
| History of CVD | 31.3 |  | 27.9 | 35.2 | 38.5 | 48.9 | <0.001 |
| Hemoglobin A_1C_, % | 8.3 (1.1) |  | 8.2 (1.0) | 8.4 (1.1) | 8.5 (1.1) | 8.5 (1.2) | <0.001 |
| Duration of diabetes, years | 9.0 (5.0-15.0) |  | 8.0 (4.5-14.0) | 10.0 (6.0-16.0) | 13.0 (7.0-20.0) | 15.0 (8.0-21.0) | <0.001 |
| Total cholesterol, mg/dL | 183.5 (41.7) |  | 182.5 (40.2) | 184.0 (44.0) | 188.1 (43.8) | 189.2 (45.1) | <0.001 |
| HDL-cholesterol, mg/dL | 41.8 (11.6) |  | 42.3 (11.5) | 41.3 (11.7) | 40.6 (11.2) | 40.1 (12.1) | <0.001 |
| LDL-cholesterol, mg/dL | 105.0 (34.0) |  | 105.1 (33.2) | 104.0 (35.2) | 107.1 (35.1) | 106.9 (35.0) | 0.132 |
| Total/HDL-cholesterol ratio | 4.7 (1.7) |  | 4.6 (1.6) | 4.8 (1.8) | 4.9 (1.6) | 5.1 (1.9) | <0.001 |
| UACR, mg/g | 14.0 (7.0-45.0) |  | 9.0 (6.0-14.0) | 56.0 (33.0-106.0) | 348.0 (58.0-649.0) | 535.5 (220.0-1066.0) | <0.001 |
| eGFR, mL/min/1.73m^2^ | 86.7 (71.8-96.7) |  | 90.4 (77.9-97.6) | 83.4 (65.9-95.9) | 66.2 (53.5-88.8) | 46.5 (40.9-52.9 | <0.001 |

^*^ P values are for comparisons between CKD risk categories using the Analysis of Variance (ANOVA) for continuous variables with a normal distribution, the Kruskal-Wallis test for continuous variables with a skewed distribution, and the χ^2^ test for categorical variables. Data are mean (standard deviation), median (interquartile range) or proportion (%) unless otherwise indicated. CKD risk categories were defined by eGFR and UACR as follows: 1) low risk, as eGFR ≥ 60 and UACR< 30; 2) moderate risk, as (45≤ eGFR <60 and UACR< 30) or (eGFR ≥ 60 and 30≤UACR≤300); 3) high risk, as (30≤ eGFR <44 and UACR< 30) or (45≤ eGFR <60 and 30≤UACR≤300) or (eGFR ≥ 60 and UACR> 300); 4) very high risk, as eGFR <30 or (30≤ eGFR <44 and 30≤UACR≤300) or (30≤ eGFR <60 and UACR>300). ACEI indicates angiotensin-converting enzyme inhibitors; ARB, angiotensin-II receptor blockers; BP, blood pressure; CKD, chronic kidney disease; CVD, cardiovascular disease; eGFR, estimated glomerular filtration rate; HDL, high-density lipoprotein; KDIGO, Kidney Disease: Improving Global Outcomes; LDL, low-density lipoprotein; UACR, urine albumin-creatinine ratio

**Supplementary Table S3. Baseline Characteristics of Participants by UACR and eGFR**

| **Characteristics** | **Whole**  **sample** |  | **UACR, mg/g** | | | |  | **eGFR, mL/min/1.73m^2^** | | |
| --- | --- | --- | --- | --- | --- | --- | --- | --- | --- | --- |
|  |  |  | **<30** | **≥30 to <300** | **≥300** | **P value^*^** |  | **≥ 60** | **< 60** | **P value^*^** |
| N | 9170 |  | 6240 | 2337 | 593 | … |  | 8211 | 959 | … |
| Age, years | 62.8 (6.6) |  | 62.5 (6.4) | 63.4 (7.0) | 62.9 (6.8) | <0.001 |  | 62.2 (6.4) | 67.4 (6.6) | <0.001 |
| Women, % | 38.2 |  | 40.5 | 32.4 | 36.8 | <0.001 |  | 37.4 | 45.4 | <0.001 |
| Race/ethnicity, % |  |  |  |  |  | <0.001 |  |  |  |  |
| *White* | 62.9 |  | 64.1 | 61.4 | 55.8 |  |  | 62.3 | 67.9 | 0.002 |
| *Black* | 18.6 |  | 17.8 | 20.5 | 21.9 |  |  | 19.3 | 14.4 |  |
| *Hispanic* | 7.0 |  | 6.8 | 7.5 | 7.3 |  |  | 7.0 | 6.9 |  |
| *Other* | 11.3 |  | 11.3 | 10.7 | 15.0 |  |  | 11.4 | 10.8 |  |
| Intensive glycemia arm, % | 50.3 |  | 50.3 | 50.7 | 48.7 | 0.692 |  | 50.1 | 51.7 | 0.347 |
| Body mass index, kg/m^2^ | 32.3 (5.4) |  | 32.2 (5.3) | 32.5 (5.5) | 32.4 (5.6) | 0.052 |  | 32.3 (5.4) | 32.3 (5.5) | 0.927 |
| Current smoking, % | 13.8 |  | 12.5 | 16.4 | 17.5 | <0.001 |  | 14.4 | 8.2 | <0.001 |
| Alcohol drinking, % | 24.4 |  | 24.6 | 25.0 | 19.6 | 0.017 |  | 25.2 | 17.2 | <0.001 |
| Systolic BP, mm Hg | 136.3 (17.0) |  | 133.6 (15.9) | 140.3 (17.1) | 148.4 (19.0) | <0.001 |  | 136.0 (16.9) | 138.6 (18.0) | <0.001 |
| Diastolic BP, mm Hg | 74.9 (10.6) |  | 74.6 (10.4) | 75.4 (11.0) | 76.7 (11.7) | <0.001 |  | 75.3 (10.5) | 71.9 (11.2) | <0.001 |
| Use of BP-lowering drug, % | 83.4 |  | 81.2 | 87.7 | 89.5 | <0.001 |  | 82.4 | 92.0 | <0.001 |
| Use of ACEI/ARB, % | 69.1 |  | 66.7 | 73.6 | 76.4 | <0.001 |  | 68.2 | 76.0 | <0.001 |
| Use of diuretics, % | 36.1 |  | 34.4 | 38.5 | 45.5 | <0.001 |  | 33.9 | 55.5 | <0.001 |
| Use of Antiplatelets/ anticoagulants, % | 57.5 |  | 57.0 | 59.1 | 55.7 | 0.144 |  | 56.9 | 62.5 | 0.001 |
| Atrial fibrillation, % | 1.2 |  | 0.8 | 2.2 | 1.7 | <0.001 |  | 1.1 | 2.7 | <0.001 |
| History of CVD | 31.3 |  | 28.8 | 35.3 | 41.7 | <0.001 |  | 30.2 | 40.2 | <0.001 |
| Hemoglobin A_1C_, % | 8.3 (1.1) |  | 8.2 (1.0) | 8.4 (1.1) | 8.6 (1.2) | <0.001 |  | 8.3 (1.1) | 8.3 (1.1) | 0.575 |
| Duration of diabetes, years | 9.0 (5.0-15.0) |  | 8.0 (5.0-14.0) | 10.0 (6.0-16.0) | 13.0 (8.0-20.0) | <0.001 |  | 9.0 (5.0-15.0) | 11.0 (6.0-19.0) | <0.001 |
| Total cholesterol, mg/dL | 183.5 (41.7) |  | 182.7 (40.3) | 183.9 (44.3) | 190.3 (44.3) | <0.001 |  | 183.3 (41.5) | 185.6 (42.8) | 0.097 |
| HDL-cholesterol, mg/dL | 41.8 (11.6) |  | 42.2 (11.5) | 41.0 (11.9) | 40.5 (10.8) | <0.001 |  | 41.9 (11.6) | 41.0 (11.4) | 0.029 |
| LDL-cholesterol, mg/dL | 105.0 (34.0) |  | 105.2 (33.3) | 103.8 (35.1) | 108.3 (35.6) | 0.013 |  | 105.0 (33.9) | 105.8 (34.7) | 0.489 |
| Total/HDL-cholesterol ratio | 4.7 (1.7) |  | 4.6 (1.6) | 4.8 (1.8) | 5.0 (1.6) | <0.001 |  | 4.7 (1.7) | 4.8 (1.7) | 0.002 |

^*^ P values are for comparisons between CKD stages using the Analysis of Variance (ANOVA) for continuous variables with a normal distribution, the Kruskal-Wallis test for continuous variables with a skewed distribution, and the χ^2^ test for categorical variables. Data are mean (standard deviation), median (interquartile range), or proportion (%) unless otherwise indicated. ACEI indicates angiotensin-converting enzyme inhibitors; ARB, angiotensin-II receptor blockers; BP, blood pressure; CVD, cardiovascular disease; eGFR, estimated glomerular filtration rate; HDL, high-density lipoprotein; LDL, low-density lipoprotein; UACR, urine albumin-creatinine ratio.

**Supplementary Table S4. Hazard ratios of incident stroke by combined measures of kidney function after Exclusion of Participants on Fenofibrate (N=** **6,687)**

| **Measures of kidney function** | | **Model 1** | |  | **Model 2** | |  | **Model 3** | |
| --- | --- | --- | --- | --- | --- | --- | --- | --- | --- |
|  |  | **HR (95% CI)** | ***P value*** |  | **HR (95% CI)** | ***P*** |  | **HR (95% CI)** | ***P*** |
| **UACR** | **eGFR** |  |  |  |  |  |  |  |  |
| **< 30** | **≥ 60** | 1 (Reference) |  |  | 1 (Reference) |  |  | 1 (Reference) |  |
| **< 30** | **<60** | 1.82 (0.90-3.66) | 0.093 |  | 1.70 (0.84-3.47) | 0.141 |  | 1.65 (0.81-3.36) | 0.170 |
| **≥ 30** | **≥ 60** | 2.19 (1.46-3.30) | <0.001 |  | 1.80 (1.18-2.76) | 0.007 |  | 1.79 (1.17-2.74) | 0.008 |
| **≥ 30** | **< 60** | 2.91 (1.51-5.59) | 0.001 |  | 2.22 (1.10-4.48) | 0.025 |  | 2.13 (1.06-4.31) | 0.034 |

Model 1 adjusted for age, sex, race and treatment arm; model 2, model 1 variables plus duration of diabetes, hemoglobin A_1C_, cigarette smoking, alcohol intake; body mass index, total-to-HDL cholesterol, systolic BP, use of BP-lowering medications, atrial fibrillation; history of CVD (excluding stroke) at baseline; model 3, model 2 variables plus use of antiplatelet agents (including aspirin)/ anticoagulants, diuretics, ACEI/ARB.

ACEI indicates angiotensin-converting enzyme inhibitors; ARB, angiotensin-II receptor blockers; BP, blood pressure; CI, confidence interval; CVD, cardiovascular disease; eGFR, estimated glomerular filtration rate; HDL, high-density lipoprotein; HR, hazard ratio; UACR, urine albumin-creatinine ratio.

**Supplementary Table S5. Hazard ratios of incident stroke by KDIGO CKD Categories after Exclusion of Participants on Fenofibrate (N=6,687)**

| **CKD Classification** | **Model 1** | |  | **Model 2** | |  | **Model 3** | |
| --- | --- | --- | --- | --- | --- | --- | --- | --- |
|  | **HR (95% CI)** | ***P value*** |  | **HR (95% CI)** | ***P value*** |  | **HR (95% CI)** | ***P value*** |
| **CKD stage** |  |  |  |  |  |  |  |  |
| ***No CKD*** | 1 (Reference) | … |  | 1 (Reference) | **…** |  | 1 (Reference) |  |
| ***CKD G1*** | 1.95 (1.14-3.33) | 0.015 |  | 1.66 (0.96-2.87) | 0.072 |  | 1.65 (0.95-2.85) | 0.076 |
| ***CKD G2*** | 2.41 (1.49-3.91) | <0.001 |  | 1.92 (1.16-3.18) | 0.012 |  | 1.89 (1.14-3.14) | 0.013 |
| ***CKD G3*** | 2.32 (1.36-3.94) | 0.002 |  | 1.96 (1.12-3.41) | 0.018 |  | 1.89 (1.08-3.30) | 0.026 |
| **CKD risk categories** |  |  |  |  |  |  |  |  |
| ***Low risk*** | 1 (Reference) | … |  | 1 (Reference) | **…** |  | 1 (Reference) |  |
| ***Moderate risk*** | 1.78 (1.16-2.72) | 0.008 |  | 1.58 (1.02-2.45) | 0.040 |  | 1.56 (1.01-2.42) | 0.045 |
| ***High risk*** | 3.45 (2.09-5.72) | <0.001 |  | 2.56 (1.49-4.43) | 0.001 |  | 2.49 (1.44-4.31) | 0.001 |
| ***Very high risk*** | 3.30 (1.40-7.81) | 0.007 |  | 2.53 (1.04-6.15) | 0.041 |  | 2.43 (1.00-5.94) | 0.051 |

CKD was classified according to the KDIGO clinical practice guidelines as follows: 1) No CKD defined as eGFR≥60 and UACR<30; 2) CKD G1, as eGFR≥90 and UACR≥ 30; 3) CKD G2, as eGFR between 60 and 89 and UACR≥30; 4) CKD G3, as eGFR between 30 and 59 regardless of UACR.

CKD risk categories were defined by eGFR and UACR as follows: 1) low risk, as eGFR ≥ 60 and UACR< 30; 2) moderate risk, as (45≤ eGFR <60 and UACR< 30) or (eGFR ≥ 60 and 30≤UACR≤300); 3) high risk, as (30≤ eGFR <44 and UACR< 30) or (45≤ eGFR <60 and 30≤UACR≤300) or (eGFR ≥ 60 and UACR> 300); 4) very high risk, as (30≤ eGFR <44 and 30≤UACR≤300) or (30≤ eGFR <60 and UACR>300).

Model 1 adjusted for age, sex, race and treatment arm; model 2, model 1 variables plus duration of diabetes, hemoglobin A_1C_, cigarette smoking, alcohol intake; body mass index, total-to-HDL cholesterol, systolic BP, use of BP-lowering medications, atrial fibrillation; history of CVD (excluding stroke) at baseline; model 3, model 2 variables plus use of antiplatelet agents (including aspirin)/ anticoagulants, diuretics, ACEI/ARB.

ACEI indicates angiotensin-converting enzyme inhibitors; ARB, angiotensin-II receptor blockers; BP, blood pressure; CKD, chronic kidney disease; CI, confidence interval; CVD, cardiovascular disease; eGFR, estimated glomerular filtration rate; HDL, high-density lipoprotein; HR, hazard ratio; KDIGO, Kidney Disease: Improving Global Outcomes; UACR, urine albumin-creatinine ratio.
